# Additive Dose Response Models: Explicit Formulation and the Loewe Additivity Consistency Condition

**DOI:** 10.3389/fphar.2018.00031

**Published:** 2018-02-06

**Authors:** Simone Lederer, Tjeerd M. H. Dijkstra, Tom Heskes

**Affiliations:** ^1^Institute for Computing and Information Sciences, Radboud University, Nijmegen, Netherlands; ^2^Max Planck Institute for Developmental Biology, Tübingen, Germany; ^3^Center for Integrative Neuroscience, University Tübingen, Tübingen, Germany

**Keywords:** dose equivalence, explicit mean equation, general isobole equation, Hill curve, null reference model, response surface, synergy

## Abstract

High-throughput techniques allow for massive screening of drug combinations. To find combinations that exhibit an interaction effect, one filters for promising compound combinations by comparing to a response without interaction. A common principle for no interaction is Loewe Additivity which is based on the assumption that no compound interacts with itself and that two doses from different compounds having the same effect are equivalent. It then should not matter whether a component is replaced by the other or vice versa. We call this assumption the Loewe Additivity Consistency Condition (LACC). We derive explicit and implicit null reference models from the Loewe Additivity principle that are equivalent when the LACC holds. Of these two formulations, the implicit formulation is the known General Isobole Equation (Loewe, 1928), whereas the explicit one is the novel contribution. The LACC is violated in a significant number of cases. In this scenario the models make different predictions. We analyze two data sets of drug screening that are non-interactive (Cokol et al., 2011; Yadav et al., 2015) and show that the LACC is mostly violated and Loewe Additivity not defined. Further, we compare the measurements of the non-interactive cases of both data sets to the theoretical null reference models in terms of bias and mean squared error. We demonstrate that the explicit formulation of the null reference model leads to smaller mean squared errors than the implicit one and is much faster to compute.

## 1. Introduction

In mixture toxicology and compound interaction modeling one is interested in synergistic or antagonistic effects between biological compounds. When combining two or more compounds, their combined effect can be much larger than the individual effects. Such a so-called synergistic effect allows for administration of lower doses to reach the same effect. This has applications in many areas such as chemotherapy (Lehar et al., 2009).

The basic understanding of synergy is any effect greater than the expected effect with no interaction assumed. This expected effect without interaction is specified with a so-called null reference model. Therefore, synergy depends highly on such a reference model of a non-interactive scenario. The central problem of defining such null reference models is the prediction of a response surface from the conditional responses. Conditional responses are the responses to a single compound, that is, conditional on the concentration of the other compound being zero.

Throughout the last century, several models for the null reference and methods to measure the deviance from these have been proposed. An extensive overview is given by Greco et al. (1995) and recent reviews are given by Geary (2012) and Foucquier and Guedj (2015). One of the most famous null reference models is the general isobole equation, which was introduced by Loewe (1928), and is based on the so-called Loewe Additivity principle. Several other models have been introduced such as Bliss Independence (Bliss, 1939), Chou and Talalay's method (Chou and Talalay, 1977), which concentrate on the null reference model locally, and the ZIP model (Yadav et al., 2015). Despite the variety of null reference models, there is no agreement on a best model or a best practice on how specifically synergy is detected. However, Loewe Additivity enjoys a wide reputation because of its principle of the sham combination. This principle rests on the idea that a compound combined with itself should yield no interaction effect.

Loewe Additivity is a phenomenological description, not a mechanistic one that is aiming to explain underlying mechanisms. This has its advantages in clinical trials, as a measure of success, such as synergy, does not need to be updated with biological advances (Fitzgerald et al., 2006). A way to root Loewe Additivity in such mechanistic terms is undertaken by Baeder et al. (2016). Further, we do not take temporal effects into consideration but work uniquely in the concentration space. While temporal considerations are important, in most high-throughput studies the effect is measured after a fixed period when transient responses have died out, but before effects like cell division set in.

We first give a short introduction to conditional dose response curves in section 2.1, to then describe the most common null reference principle, Loewe Additivity. We study Loewe Additivity's consistency condition and its consequences in section 2.2. Further, in section 2.3 we introduce an explicit null reference model derived from the Loewe Additivity principle, which describes the same null reference model as the general isobole equation, when the Loewe Additivity consistency condition is met. As this consistency condition is often violated by experimental data (Geary, 2012; Tallarida, 2012) we investigate the consequences of these violations to the null reference models visually at the end of section 2.3, and evaluate them in section 3.

## 2. Materials and methods

### 2.1. Introduction and background

As the first experiments for the assessment of synergy were conducted *in vivo*, one used to administer varying doses of compounds, that is, the unit of compound per kilogram of biological system under investigation. That historical term still remains in the research area of synergy and often the term dose is used to actually refer to concentrations, the number of molecules per unit volume. In this study, we refer to the response as plotted on the y-axis of a dose-response curve as response. Both of our examples on data are inhibitory, where the response consists of cell survival. Thus a larger dose leads to less cells surviving and hence a smaller response. We use effect to denote the inverse of response. Thus, for dose zero we have maximal response and minimal effect and for infinite dose we have minimal response and maximal effect. In the literature this measured effect is also referred to as the phenotypic effect or as cell survival of disease agents or cancer cell lines. Measurements taken for only one compound, here referred to as the conditional responses or individual dose response, are also called mono-therapeutic (Di Veroli et al., 2016) or single compound, but we prefer a more statistical terminology. We refer to the measurements of one cell line exposed to all combinations of the two compounds as a record, but in other literature it is referred to as response matrix (Lehár et al., 2007; Yadav et al., 2015).

To quantify the degree of synergy between two compounds, the typical approach is to somehow compare their measured combination effect to a so-called null reference model: the expected response assuming no interaction between the two compounds. The larger the deviance to such a null reference model, the larger the interaction effect. Writing *x*_*j*_ for the dose of compound *j* ∈ {1, 2}, a null reference model specifies the response *f*(*x*_1_, *x*_2_) for a combination of doses *x*_1_ and *x*_2_. In the next few sections, we will first review the ideas leading to a specific null reference model, the so-called general isobole equation, which is based on the principle of Loewe Additivity. Our own contribution starts with section 2.2, in which we write down the precise assumption underlying this principle and discuss its consequences.

#### 2.1.1. Conditional individual dose response curves

**Figure 1:**
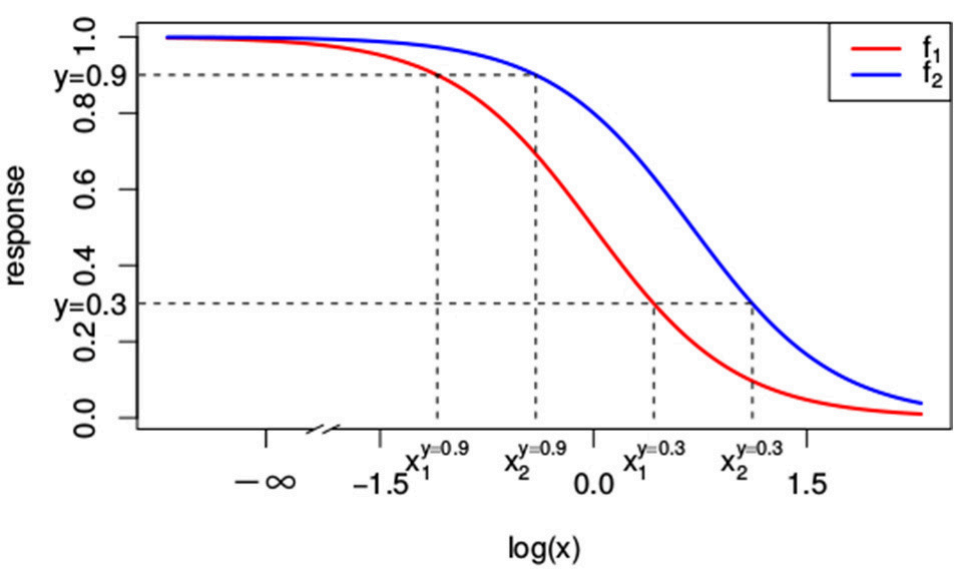
Two different Hill curves (red and blue). Dashed lines indicate the different doses *x*_1_ and *x*_2_ that reach a response of 0.3 and 0.9. The dose-response curves differ only in EC50 with *e*_1_ = 1 and *e*_2_ = 2. Values of the other parameters are *y*_0_ = 1, *y*_∞_ = 0 and *s* = 2.

As we will see, null reference models are build on top of the dose-response curves for the individual compounds. That is, the null reference model extrapolates individual dose-response curves, i.e., *f*_1_(*x*_1_) ≡ *f*(*x*_1_, 0) and *f*_2_(*x*_2_) ≡ *f*(0, *x*_2_), to a response *f*(*x*_1_, *x*_2_) for any combination of doses (*x*_1_, *x*_2_). A popular model for individual dose-response curves *f*_*j*_(*x*_*j*_) with *j* ∈ {1, 2} is the Hill curve (Hill, 1910), also referred to as the sigmoid function. The Hill model is, due to its good fit to many sources of data, the most widely applied model for fitting compound responses (Goutelle et al., 2008). It has a sigmoidal shape with little change in response for small doses, but a rapid decline once a certain threshold is met. For even larger doses the response asymptotes to a constant, which corresponds to the maximal effect. The red and blue lines in Figure 1 correspond to two different Hill curves.

There are several parameterizations of the Hill curve. In this paper, we work with the so-called four-parameter log-logistic model, which is also used in the drc package (Ritz et al., 2015):

(1) $$\begin{array}{r} f\left(x\right)=y_{\infty }+\frac{y_{0}-y_{\infty }}{1+{\left(\frac{x}{e}\right)}^{s}}, \end{array}$$

where *y*_0_ is the response at zero dose and *y*_∞_ the maximal effect of the cells to the compound, *e* the dose concentration reaching half of the maximal effect and *s* the steepness of the curve, where a positive *s* leads to a monotonically decreasing curve.

We use the Hill curves to illustrate our theory and methods and to fit individual dose-response curves to real-world data in section 3. Our theoretical analysis and the methods that result from that, are not restricted to the use of Hill curves as model for individual dose-response curves, but apply more generally to any type of dose-response model, as long as it is monotonically decreasing or increasing and twice continuously differentiable.

#### 2.1.2. Loewe additivity

Throughout the extensive research that was conducted in the field of synergy over the last century, several null reference principles were introduced, but only two survived the critics (Greco et al., 1995): Loewe Additivity (Loewe, 1928) and Bliss Independence (Bliss, 1939). Loewe Additivity assumes that one compound can be substituted for another, which makes sense when the two compounds have the same mechanism of action. In Bliss Independence, on the other hand, the underlying assumption is that the two compounds have a different mechanism of action, leading to an addition of the individual responses. In this paper, we will exclusively focus on Loewe Additivity, which tends to lead to better predictions of synergy than Bliss Independence (Cokol et al., 2011).

**Figure 2:**
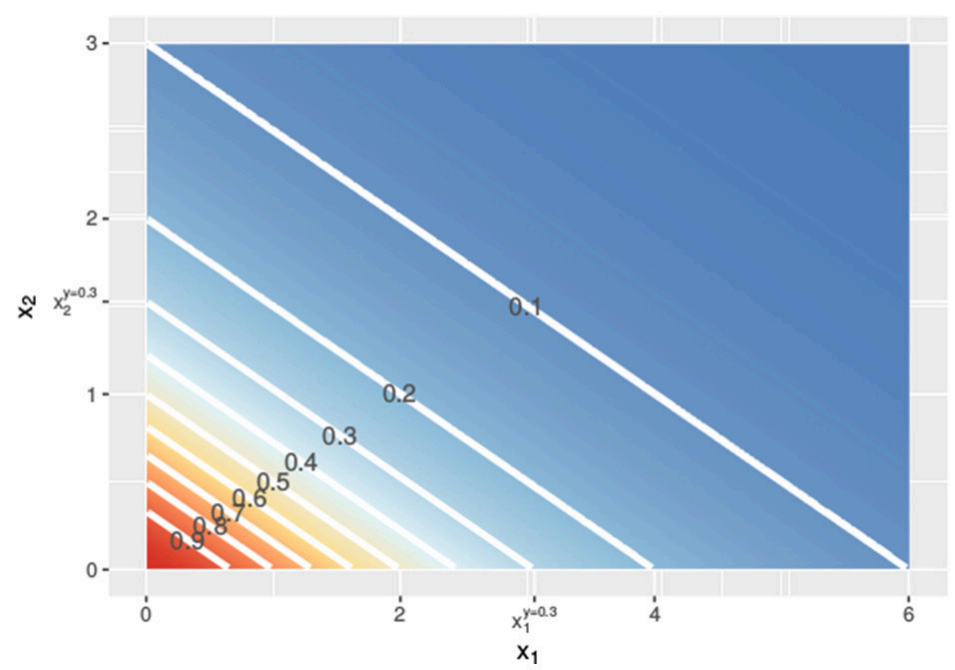
Contour lines of the response surface from Equations (3), (6), or (7) with *x*_1_ on the *x*-axis and *x*_2_ on the *y*-axis at linear concentrations.

Loewe argued that, if two individual doses $x_{1}^{*}$ and $x_{2}^{*}$ give rise to the same response, say *y*, then, in case of no interaction between the compounds, all dose combinations on the straight line running from $\left(x_{1}^{*},0\right)$ to $\left(0,x_{2}^{*}\right)$, i.e., for which

(2) $$\begin{array}{r} \frac{x_{1}}{x_{1}^{*}}+\frac{x_{2}}{x_{2}^{*}}=1, \end{array}$$

should yield the exact same response *y*. Intuitively and visually, this idea is very appealing. We illustrate this concept in Figures 1, 2.

In Figure 1, the lower dashed horizontal line corresponds to the response *y* = 0.3, e.g., representing the survival of 30% of the cell culture. The individual doses, $x_{1}^{*}$ and $x_{2}^{*}$, that yield this response follow from the intersection this horizontal line with the red and the blue individual response curves, respectively. They are indicated by the two vertical dashed lines. To visualize the response surface for arbitrary combinations of doses *x*_1_ and *x*_2_, we make use of a contour plot in a two-dimensional coordinate system, as in Figure 2, with the dose *x*_1_ along the x-axis and the dose *x*_2_ along the y-axis. Contour lines correspond to so-called isoboles or iso-effect curves: combinations of doses (*x*_1_, *x*_2_) that yield the exact same response. For *y* = 0.3, we already know two points on this isobole: $\left(x_{1}^{*},0\right)$ and $\left(0,x_{2}^{*}\right)$. Loewe Additivity now says that the isobole in case of no interaction should be linear, that is, following Equation (2).

The straight line matches the assumption that the two compounds “act similarly, presumably at the same site of action, differing only in potency” (Greco et al., 1995, p. 344). It suggests that doses for the two compounds are exchangeable, more specifically that a dose *x*_1_ which is *d%* of the dose $x_{1}^{*}$ needed to reach the response *y* by just the first compound, has the same effect as a dose *x*_2_ which is the same *d%* of the dose $x_{2}^{*}$ needed to reach the response *y* by just the second compound. This argumentation, in combination with the principle of a sham combination (two doses of the same compound must have the same effect as a single compound with the sum of the doses), directly leads to Equation (2). In section 2.2 we will get back to this argumentation and discuss in detail the properties that individual dose-response curves should have for this argumentation to stand.

#### 2.1.3. General isobole equation

In the above, we showed, following Loewe, how to find dose combinations (*x*_1_, *x*_2_) that yield the same response *y* as the two equivalent doses $x_{1}^{*}$ and $x_{2}^{*}$ that individually reach this response *y*. Applying the same procedure for different values of *y*, we get the contour lines in Figure 2 and can construct a response surface for any combination of doses (*x*_1_, *x*_2_).

The corresponding null reference model is called the general isobole equation (Loewe, 1928, p.179; Berenbaum, 1989). It is defined as *f*_GI_(*x*_1_, *x*_2_) = *y* with *y* the solution of

(3) $$\begin{array}{r} \frac{x_{1}}{f_{1}^{-1}\left(y\right)}+\frac{x_{2}}{f_{2}^{-1}\left(y\right)}=1, \end{array}$$

which corresponds to Equation (2) with substitutions $x_{1}^{*}=f_{1}^{-1}\left(y\right)$ and $x_{2}^{*}=f_{2}^{-1}\left(y\right)$. The general isobole equation defines the response curve *implicitly* and for most types of individual dose-response curves, including Hill curves, numerical computations are needed to derive *f*_GI_(*x*_1_, *x*_2_). This can be considered a practical disadvantage, in particular when applied to high-throughput data. More details on how to solve Equation (3) numerically are presented in Supplementary Material 5.

### 2.2. Theory

Above we derived the general isobole equation, an implicitly defined null reference model based on the principle of Loewe Additivity. The argumentation leading to the straight isoboles, although appealing, was rather informal, as it was in Loewe's original work (Loewe, 1928) and in most of the literature that followed. In this section we will formalize the argumentation to arrive at the conclusion that strictly adhering to the Loewe Additivity principle puts very serious constraints on the (relationship between the) individual dose-response curves of the two compounds.

#### 2.2.1. Loewe additivity consistency condition

Loewe Additivity says that we can exchange one compound for another to reach the same effect. We define the effect equivalent dose $x_{1}^{\text{equiv}}\left(x_{2}\right)$ as the dose of compound 1 that yields the same response as dose *x*_2_ of compound 2, and vice versa for $x_{2}^{\text{equiv}}\left(x_{1}\right)$. Given individual dose-response curves *f*_1_(*x*_1_) and *f*_2_(*x*_2_), these effect equivalent doses obey

(4) $$\begin{array}{r} x_{1}^{\text{equiv}}\left(x_{2}\right)=f_{1}^{-1}\left(f_{2}\left(x_{2}\right)\right) \end{array}$$

(5) $$\begin{array}{r} x_{2}^{\text{equiv}}\left(x_{1}\right)=f_{2}^{-1}\left(f_{1}\left(x_{1}\right)\right). \end{array}$$

The construction of response equivalent doses is illustrated in Figure 1 for the response levels *y* = 0.9 and *y* = 0.3.

With these equivalent doses one can construct two response surfaces. To do so, we add to the concentration *x*_1_ of compound 1 its equivalent dose $x_{1}^{\text{equiv}}\left(x_{2}\right)$ and the same mutatis mutandis for *x*_2_ and compute the response:

(6) $$\begin{array}{r} f_{2\to 1}\left(x_{1},x_{2}\right)=f_{1}\left(x_{1}+f_{1}^{-1}\left(f_{2}\left(x_{2}\right)\right)\right) \end{array}$$

(7) $$\begin{array}{r} f_{1\to 2}\left(x_{1},x_{2}\right)=f_{2}\left(f_{2}^{-1}\left(f_{1}\left(x_{1}\right)\right)+x_{2}\right). \end{array}$$

We can use both response curves as null reference models: just like the general isobole equation they specify the expected response for any combination of doses under the assumption of no interaction between the two compounds. If the individual dose-response curves are analytically invertible, as for the Hill curves that we use throughout this paper, these null reference models are *explicit* and do not require complicated numerical computations. It so happens that for our running example in Figure 1, the two response curves *f*_2→1_(*x*_1_, *x*_2_) and *f*_1→2_(*x*_1_, *x*_2_) coincide with each other and with *f*_GI_(*x*_1_, *x*_2_), leading to the exact same contour plot in Figure 2. We will see in section 2.2.2 why.

Taking the principle of Loewe Additivity seriously, it should not matter whether we exchange the dose for compound 1 with the equivalent dose for compound 2 or, vice versa, exchange the dose for compound 2 with the equivalent dose for compound 1. We formalize this in the *Loewe Additivity Consistency Condition*, further referred to as LACC:

(8) $$\begin{array}{r} f_{2\to 1}\left(x_{1},x_{2}\right)=f_{1\to 2}\left(x_{1},x_{2}\right)\;\forall x_{1},x_{2}\in {\mathbb{R}}_{\geq 0}. \end{array}$$

To the best of our knowledge, we are the first to explicitly state this consistency condition in a general mathematical form.

#### 2.2.2. Conditions for the LACC to hold

Perhaps surprisingly and in contrast with suggestions elsewhere (e.g., Greco et al., 1995; Yadav et al., 2015), the Loewe Additivity Consistency Condition is easily violated and poses strong restrictions on the relationship between the two individual dose-response curves. Specifically, we have the following theorem.

**Theorem 1**. *The Loewe Additivity Consistency Condition in Equation (8) holds, if and only if a dose and its equivalent are proportional to each other, i.e.*,

(9) $$\begin{array}{r} x_{1}^{\text{equiv}}\left(x_{2}\right)=f_{1}^{-1}\left(f_{2}\left(x_{2}\right)\right)=cx_{2}, \end{array}$$

(10) $$\begin{array}{r} x_{2}^{\text{equiv}}\left(x_{1}\right)=f_{2}^{-1}\left(f_{1}\left(x_{1}\right)\right)=\frac{1}{c}x_{1}, \end{array}$$

*for a constant c > 0*.

The proof of this theorem can be found in Supplementary Material 1. Both Tallarida (2012) and Geary (2012) recently commented on the connection between the consistency condition in Equation (8) and the proportionality between a dose and its equivalent, but did not provide a theoretical proof. Note that in the proof and in the following discussion of the LACC, we make the implicit assumption that both response curves start off at the same response for zero dose and yield the same maximal effects, i.e., converge to the same asymptotes when the dose goes to infinity. If not, there are doses for one compound that do not have an equivalent dose for the other. In section 2.3.2 we discuss how to adapt the null reference models if this condition is not met.

Rewriting Equation (9), we have

(11) $$\begin{array}{r} \log\left(x_{1}^{\text{equiv}}\left(x_{2}\right)\right)=\log\left(c\right)+\log\left(x_{2}\right), \end{array}$$

i.e., the LACC holds if and only if the dose-response curves are shifted copies of each other on the logarithmic dose axis. If the individual dose-response curves for the two compounds are Hill curves, this implies that only the dose concentration reaching half of the maximal effect (*e* in Equation 1) can differ: both Hill curves should have the same responses at zero dose (*y*_0_), the same maximal effect (*y*_∞_), and the same slopes (*s*) for the LACC to hold (see Supplementary Material 3). Since the two Hill curves in Figure 1 are indeed shifted horizontally relative to each other with the same *y*_0_, *y*_∞_, and *s*, yet different *e*, in this case the LACC indeed holds. The implicit general isobole equation from Equation (3) and the explicit null reference models from Equations (6, 7) all yield the same response surface and all isoboles in Figure 2 are linear and parallel to each other.

This equivalence and the parallel linear isoboles are not a coincidence, as can be seen from the following corollary to Theorem 1:

**Corollary 1**. *If the Loewe Additivity Consistency Condition in Equation (8) holds, (1) f_GI_(x_1_, x_2_) = f_2→1_(x_1_, x_2_) = f_1→2_(x_1_, x_2_) and (2) the isoboles corresponding to these models are parallel*.

The proof of this corollary can be found in Supplementary Material 2.

### 2.3. Methods

Our running example in Figures 1, 2 represents the exception rather than the rule. As we have seen, the LACC requires a very specific interplay between the two individual dose-response curves, which is likely often violated in practice. This sheds doubts on the value of an implicit formulation of a response surface as the one provided by the general isobole equation for real-world applications. If the LACC does hold, *f*_GI_(*x*_1_, *x*_2_) is equivalent to explicit formulations such as *f*_2→1_(*x*_1_, *x*_2_) and *f*_1→2_(*x*_1_, *x*_2_) that are much easier to compute. If the LACC does not hold, we may still for aesthetic reasons prefer linear isoboles over nonlinear ones (explained in section 2.3.3), but the precise argumentation that leads to these linear isoboles breaks down.

So, apart from aesthetic and perhaps historical reasons, the relevant question is whether an implicit formulation as general isobole equation leads in practice, when the LACC is violated, to better fitting response surfaces than similar explicit formulations. This will be investigated in section 3. In this section, we will derive a symmetric explicit null reference model that, for mild violations of the LACC, still gives close to linear isoboles. We will adapt the different null reference models to handle cases in which the two dose-response curves have different maximal effects and will then illustrate their response surfaces.

#### 2.3.1. Explicit mean equation

Unlike the general isobole equation, the two explicit models *f*_2→1_(*x*_1_, *x*_2_) and *f*_1→2_(*x*_1_, *x*_2_) are asymmetric, that is, the two compounds cannot be interchanged without leading to different results when the LACC does not hold. A simple remedy is to consider a weighted combination of both, as in

(12) $$\begin{array}{l} f_{\text{mean}}(x_{1},x_{2})=\beta (x_{1},x_{2})f_{2\to 1}(x_{1},x_{2})\\ \;+[1-\beta (x_{1},x_{2})]f_{1\to 2}(x_{1},x_{2}), \end{array}$$

with β(*x*_1_, *x*_2_) ∈ [0, 1] and β(*x*_1_, *x*_2_) = 1−β(*x*_2_, *x*_1_) to enforce symmetry. Obviously, when the LACC holds, we still have *f*_mean_(*x*1, *x*2) = *f*_GI_(*x*_1_, *x*_2_). We aim for a choice of β(*x*_1_, *x*_2_) such that under mild violations of the LACC, the explicit formulation is still close to the implicit one, i.e., *f*_mean_(*x*_1_, *x*_2_) ≈ *f*_GI_(*x*_1_, *x*_2_), which then also will result in isoboles that are still close to linear.

In Supplementary Material 4, we prove that a simple arithmetic mean,

(13) $$\begin{array}{c} f_{\text{mean}}(x_{1},x_{2})=\frac{1}{2}[f_{2\to 1}(x_{1},x_{2})+f_{1\to 2}(x_{1},x_{2})], \end{array}$$

does the best job: for mild violations of the LACC it stays close to the general isobole equation and hence may be an alternative worth investigating. In the following, we will refer to Equation (13) as the explicit mean equation. A geometric mean instead of a simple arithmetic mean also closely matches the general isobole equation for mild violations of the LACC. Details can be found in Supplementary Material 7.

#### 2.3.2. Different maximal effects

In case one dose reaches an effect that cannot be reached by the other, there is no equivalence relationship between the two doses and therefore Loewe Additivity cannot hold. Here we discuss how to adapt the general isobole equation and the explicit mean equation to this situation.

Let us first assume that dose *x*_2_ of compound 2 leads to an effect that cannot be reached by any dose *x*_1_ of compound 1:

(14) $$\begin{array}{r} f_{2}\left(x_{2}\right)<\underset{x_{1}}{\min}f_{1}\left(x_{1}\right). \end{array}$$

**Figure 3:**
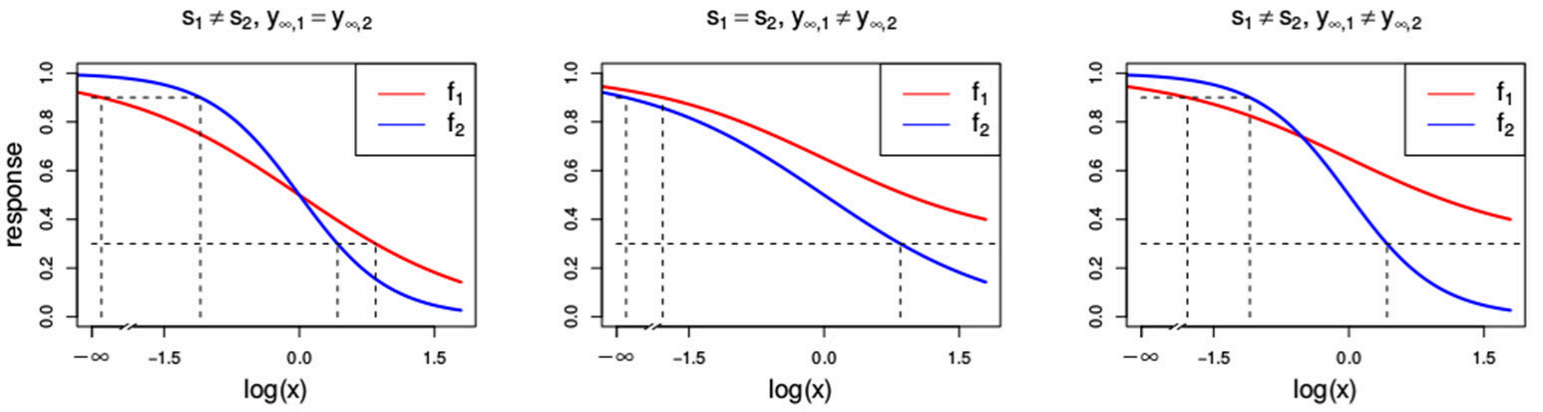
Dose-response curves (red and blue) with different parameter settings that all violate the LACC: *s*_1_ ≠ *s*_2_ **(Left)** with *s*_1_ = 1 and *s*_2_ = 2, *y*_∞, 1_ ≠ *y*_∞, 2_ **(Center)** with *y*_∞, 1_ = 0.3 and *y*_∞, 2_ = 0, and **(Right)** with *s*_1_ ≠ *s*_2_ and *y*_∞, 1_ ≠ *y*_∞, 2_ with the same settings as above. Additionally, all other parameters that are chosen to be equal for both Hill curves take the values *s* = 1, *y*_0_ = 1, *y*_∞_ = 0 and *e* = 1.

This is depicted in Figure 3 in the middle and right panel, where the first compound reaches a maximal effect of *y*_∞, 1_ = 0.3 and the second one a maximal effect of *y*_∞, 2_ = 0. For the general isobole equation, Di Veroli et al. (2016) suggests that one would need an infinite dose of *x*_1_ to yield a response as close as possible to *f*_1_(*x*_1_), and hence proposes to set the first term in Equation (3) to zero to arrive at

(15) $$\begin{array}{r} \frac{x_{2}}{f_{2}^{-1}\left(y\right)}=1, \end{array}$$

with the obvious solution *f*_GI_(*x*_1_, *x*_2_) = *f*_2_(*x*_2_). The other way around, we set *f*_GI_(*x*_1_, *x*_2_) = *f*_1_(*x*_1_) when *f*_1_(*x*_1_) < min_*x*_2__*f*_2_(*x*_2_).

Following a similar line of reasoning as in Di Veroli et al. (2016), we propose

(16) $$\begin{array}{l} f_{2\to 1}\left(x_{1},x_{2}\right)=f_{1}\left(x_{1}+x_{1}^{\text{equiv}}\left(x_{2}\right)\right)=f_{1}\left(x_{1}^{\text{equiv}}\left(x_{2}\right)\right)\\ \;=f_{1}\left(f_{1}^{-1}\left(f_{2}\left(x_{2}\right)\right)\right)=f_{2}\left(x_{2}\right), \end{array}$$

for the case *f*_2_(*x*_2_) < min_*x*_1__*f*_1_(*x*_1_), where the second step follows since $x_{1}^{\text{equiv}}\left(x_{2}\right)$ dominates *x*_1_. Note that in this case *f*_2→1_(*x*_1_, *x*_2_) still follows from its original definition in Equation (6). Similarly, when $f_{1}\left(x_{1}\right)<\underset{x_{2}}{\min}f_{2}\left(x_{2}\right)$, we set *f*_1→2_(*x*_1_, *x*_2_) = *f*_1_(*x*_1_) and leave *f*_2→1_(*x*_1_, *x*_2_) unchanged. In both cases, the explicit mean equation still equals the average of *f*_2→1_(*x*_1_, *x*_2_) and *f*_1→2_(*x*_1_, *x*_2_), which, in case of *f*_2_(*x*_2_) < min_*x*_1__*f*_1_(*x*_1_):

(17) $$\begin{array}{c} f_{\text{mean}}(x_{1},x_{2})=\frac{1}{2}[f_{2}(x_{2})+f_{2}(f_{2}^{-1}(f_{1}(x_{1}))+x_{2})]. \end{array}$$

Note that this is different to the general isobole equation, which takes the form *f*_GI_(*x*_1_, *x*_2_) = *f*_2_(*x*_2_).

#### 2.3.3. Illustrations of violations of LACC

As mentioned before and commented by Tallarida (2012) and Geary (2012), the conditional response curves of experimental data are often not proportional and therefore, the LACC in Equation (8) is often violated. Here, we investigate what different violations of the LACC imply for the null reference models.

**Figure 4:**
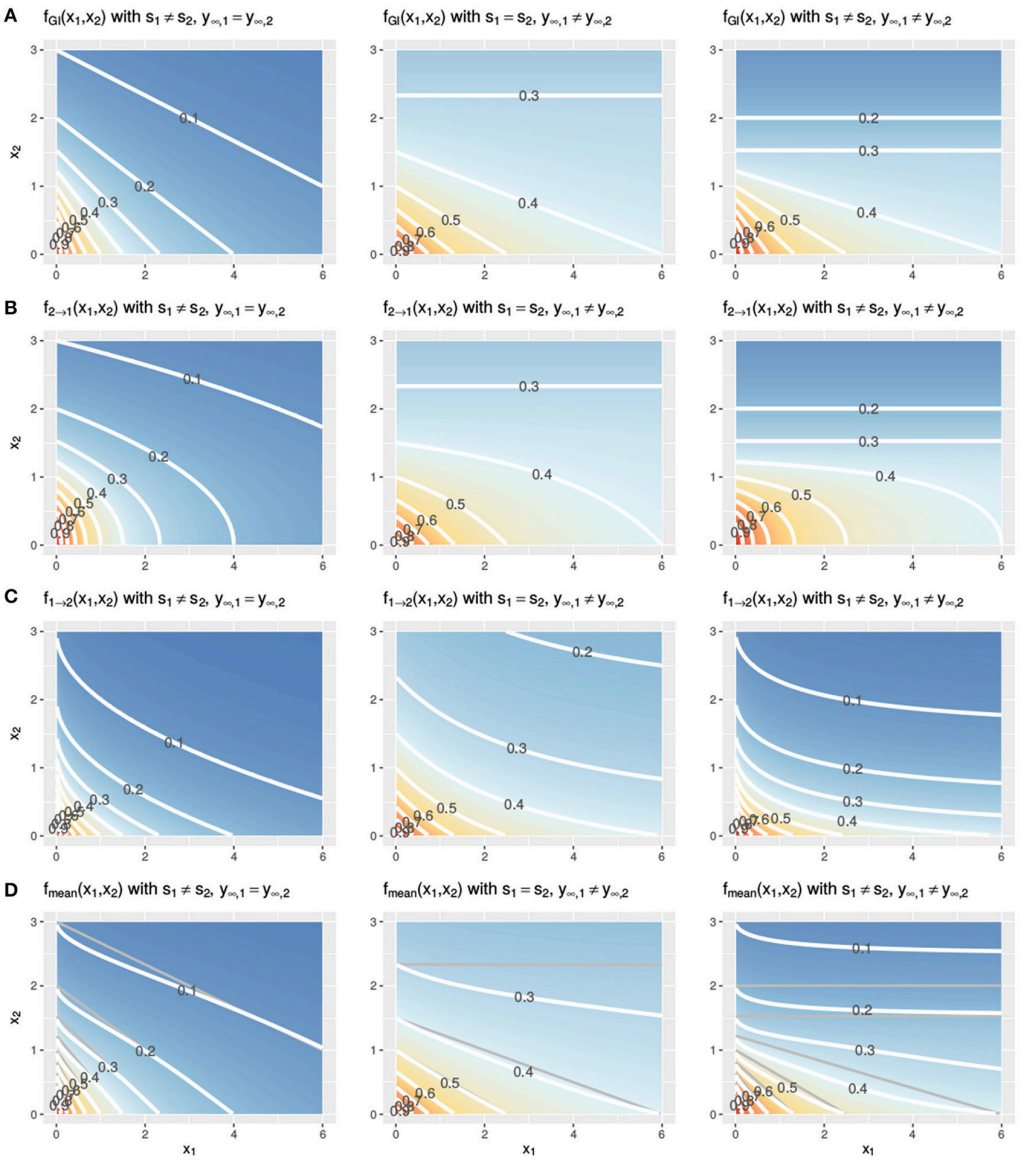
Three cases of violation of the LACC for the *f*_GI_(*x*_1_, *x*_2_) **(A)**, *f*_2→1_(*x*1, *x*2) **(B)**, *f*_1→2_(*x*1, *x*2) **(C)**, and *f*_mean_(*x*_1_, *x*_2_) **(D)** model. Contour lines are depicted in white. In **(D)** the contour lines of the corresponding *f*_GI_(*x*_1_, *x*_2_) are depicted in gray for reference. The parameter setting is the following from left to right: (left) *s*_1_ ≠ *s*_2_, here depicted with *s*_1_ = 1, *s*_2_ = 2, *y*_∞_ = 0, or (middle) the maximal effect values differ, *y*_∞, 1_ ≠ *y*_∞, 2_, here shown with *s* = 1, *y*_∞, 1_ = 0.3, *y*_∞, 2_ = 0 or (right) both, the slopes and the maximal effects are different, here shown with *s*_1_ = 1, *s*_2_ = 2, *y*_∞, 1_ = 0.3, *y*_∞, 2_ = 0. The remaining two parameters of the Hill curve are set equal for all figures to *y*_0_ = 1 and *e* = 1.

In Figure 3, two Hill curves are depicted in three scenarios where the LACC is violated: two different slopes *s* (left) or two different maximal effects *y*_∞_ (middle) or both (right). It becomes immediately clear that in all scenarios there is no proportional relationship between the two curves. Figure 4 shows the three null reference models *f*_GI_(*x*_1_, *x*_2_), *f*_2→1_(*x*1, *x*2), *f*_1→2_(*x*1, *x*2) and *f*_mean_(*x*_1_, *x*_2_) with the Hill curves from Figure 3. Figure 4A depicts *f*_GI_(*x*_1_, *x*_2_) model while the three explicit models, *f*_2→1_(*x*_1_, *x*_2_) and *f*_1→2_(*x*_1_, *x*_2_) from Equations (6, 7) and *f*_mean_(*x*_1_, *x*_2_) from Equation (13), are depicted in Figures 4B–D, respectively. In each column, the three cases of LACC violation (Figure 3 are depicted). The contour lines of the *f*_mean_(*x*_1_, *x*_2_) in Figure 4D are depicted in white and for reference, the contour lines of *f*_GI_(*x*_1_, *x*_2_) are depicted in gray.

Let us first investigate in detail the first case of violation, assuming different slope parameters for the conditional responses, as depicted in the left column of Figure 4. The *f*_GI_(*x*_1_, *x*_2_) displays straight isoboles, which are not parallel as they are in Figure 2. The straightness is due to Berenbaum's definition of the general isobole equation and becomes obvious by inspection of Equation (3), which is symmetric in the fractional terms. This is one of the reasons why this model has been popular. The two explicit models in the left panel of Figures 4B,C display a concave or convex curvature to the point of zero dose concentration. The explicit mean equation in Figure 4D shows nearly linear isoboles, with a slight curvature which is almost linear for *x*_1_ reaching a larger effect than *x*_2_ and convex for *x*_1_ reaching a smaller effect than *x*_2_.

The scenario for different maximal effects but the same slopes is depicted the middle panels of Figure 4 for the *f*_GI_(*x*_1_, *x*_2_) model as well as for the explicit models *f*_2→1_(*x*1, *x*2), *f*_1→2_(*x*1, *x*2) and *f*_mean_(*x*_1_, *x*_2_). The models all have a smaller effect than in the previous scenario, where the slopes differ. All explicit models exhibit nonlinear isoboles. *f*_2→1_(*x*1, *x*2) and *f*_1→2_(*x*1, *x*2) exhibit a similar concave and convex curvature behavior as in the scenario of differing slopes. For *f*_GI_(*x*_1_, *x*_2_), *f*_2→1_(*x*1, *x*2) and *f*_mean_(*x*_1_, *x*_2_), the asymptotic behavior is depicted. For *f*_GI_(*x*_1_, *x*_2_) and *f*_2→1_(*x*1, *x*2), these figures display a constant horizontal response for *x*_1_, only decreasing for increasing *x*_2_ doses. The isoboles of *f*_mean_(*x*_1_, *x*_2_), which is formed by the mean of *f*_2→1_(*x*1, *x*2) and *f*_1→2_(*x*1, *x*2), are convex for small doses of *x*_1_ and then become almost linear for increasing doses of *x*_1_.

For the case where both scenarios of a violation are met, namely different slopes and different maximal effects, we depict the response surfaces of the null models in the right column of Figure 4. Here, we see the characteristics from the two violations combined, namely, non-linear isoboles for the explicit null reference model, but *f*_mean_(*x*_1_, *x*_2_) exhibiting almost linear isoboles, together with an asymptotic effect behavior for Figures 4A,B.

### 2.4. Material

Two data sets are used throughout this research: The first data set was created by Mathews Griner et al. (2014) and is a cancer compound synergy study. We refer to this data set as the Mathews Griner data. It is composed of 463 different drug-drug-cell combinations on the cancer cell line TMD8 and was published along with many other large compound-drug-cell combination studies on the website https://tripod.nih.gov/matrix-client/. It is a so-called one-to-all experiment, meaning that one compound (in this case ibrutinib) is combined with 463 other compounds. In this high-throughput study all 463 compound combinations are screened in a 6 × 6 matrix design and the effect of the compound combinations is measured as cell viability. The six different concentrations of ibrutinib and the paired compound decrease from 2.5 and 125μM four times with a 4-fold dilution with the sixth dose being zero (Yadav et al., 2015).

In a synergy modeling study, Yadav et al. (2015) categorized each record of the Mathews Griner data into three interaction classes after visual inspection of its dose-response matrix: synergy, no interaction, antagonism. We only use the 252 dose response matrices classified as non-interactive.

The other data set used in this study with a labeling of the records is the anti fungal cell growth experiment on the yeast *S. cerevisiae* (strain BY4741) by Cokol et al. (2011) and from here on referred to as the Cokol data set. In this study 200 different drug-drug-cell combinations were conducted with 33 different compounds and growth inhibition was measured. An 8 × 8 factorial design is used with doses linearly increasing from 0 up to a dose close to the individually measured maximal effect dose of the compound under investigation.

The categorization of this data set is based on a comparison of the longest arc length of an isobole relative to the expected longest linear isobole in a non-interactive scenario. In more detail, having estimated the response surface of a record, Cokol et al. (2011) chose the longest contour line and measure its length and direction (convex or concave). In case of the contour line being convex the record is categorized as synergistic and the arc length of the longest contour line determines the strength of synergy. As the labeling of these records is quantitative, we consider all records to be non-interactive if their absolute value is smaller than 0.8. This decision is based on communication with the authors (Cokol et al., 2011). This leaves us with 82 records.

We received both categorizations after personal communication with the authors (Cokol et al., 2011; Yadav et al., 2015). For the purpose of comparing the null reference models introduced in sections 2.1 and 2.3, we consider these two classifications as ground truth, given that no molecular information is available for verification.

The conditional responses are fitted with Hill curves individually, with the constraint to share the *y*_0_ parameter. For the fitting, we make use of the drc package (Ritz et al., 2015). More detailed information is given in Supplementary Material 5. Records with negative slopes or negative EC50 values are excluded, which leaves us with 159 records for the Mathews Griner and 79 for the Cokol data. The main reason for the exclusion of nearly 40% of the records of the Mathews Griner data is the fixed dose range applied to all compounds. Many conditional readouts show barely any response over the entire dose range. The compounds might therefore have no effect at all on the cell line or the dose range is too small to cause any effect.

From the fitted conditional responses, we construct two response surfaces, one for the general isobole equation and one for the explicit mean equation. Residuals are calculated by subtracting from each observed response (maximally 36 values for Mathews Griner and 64 for Cokol) the predicted responses from the General Isobole and Explicit Mean response surfaces. We summarize the residuals for each response matrix by their mean and standard deviation. These capture the bias and mean squared error, respectively.

## 3. Results

With the two data sets introduced in section 2.4 we confirm Geary's statement about the common violation of the LACC. Furthermore, we compare the different null reference models by considering non-interactive records of two different data sets of compound screenings.

### 3.1. Violation of the LACC

To support the statement from section 2.3 about the LACC being often violated, we apply Wilcoxon signed-rank test to the Mathews Griner data which combines one compound with a set of other compounds. The first test checks the null hypothesis that the slopes *s* from the two fitted conditional responses are equal. The second tests for equality of the maximal effects *y*_∞_. The results of both tests on *s* and *y*_∞_ are significant with $p_{s}=6.26\times 10^{-5}$ and $p_{y_{\infty }}=2.2\times 10^{-16}$, respectively. Thus, the LACC is often violated due to differing slopes *s* and maximal effects *y*_∞_.

### 3.2. Quality of fit

We compare *f*_GI_(*x*_1_, *x*_2_) (Equation 3) and *f*_mean_(*x*_1_, *x*_2_) (Equation 13) by computing the bias and mean squared error between the null reference surfaces that are spanned by the null reference models and the measured response data, excluding the outliers (see Supplementary Material 5), namely

(18) $$\begin{array}{r} {\text{bias}}_{j}=\frac{1}{N}\overset{N}{\underset{i=1}{\sum }}\left({ŷ}_{i,j}-y_{i,j}\right) \end{array}$$

(19) $$\begin{array}{r} {\text{mse}}_{j}=\frac{1}{N}\overset{N}{\underset{i=1}{\sum }}{\left({ŷ}_{i,j}-y_{i,j}\right)}^{2} \end{array}$$

with *ŷ*_*i, j*_ being the estimated response and *y*_*i, j*_ the measured response for dose combination *i* and record *j*, and mse short for mean squared error.

**Figure 5:**
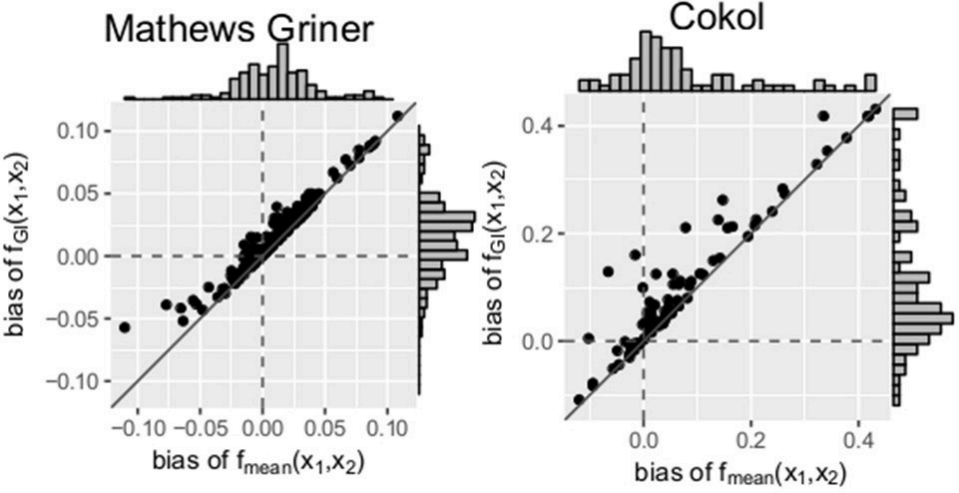
Bias: mean difference between the responses given by the model and the measured responses. To better qualify the differences in bias, the diagonal is depicted. The distribution of the models' bias values is given in histograms plotted on the axes.

Scatter plots of the bias of both data sets are depicted in Figure 5. For every record, the bias of *f*_mean_(*x*_1_, *x*_2_) is depicted on the *x*- and the bias of *f*_GI_(*x*_1_, *x*_2_) on the *y*-axis. A striking observation from Figure 5 is that the bias values of *f*_GI_(*x*_1_, *x*_2_) are always larger than those of *f*_mean_(*x*_1_, *x*_2_). This holds for both data sets. For positive bias values, this gives a smaller bias for *f*_mean_(*x*_1_, *x*_2_) and for negative bias values, a smaller bias in absolute terms for *f*_GI_(*x*_1_, *x*_2_). We look in detail into the bias, namely the individual differences of estimated data points to the measured ones, for each record. For all records of the Mathews Griner and most records for the Cokol data set, the residuals of each data point, meaning the read-out for a given dose combination, are larger for the *f*_GI_(*x*_1_, *x*_2_) model. We suspect this to be due to the definition of the explicit models, as, by taking into consideration the effect of the other compound as well, the spanned surfaces are steeper decreasing if the LACC is violated. This becomes clear by inspecting the right panel of Figure 4D, for which the contour lines of the *f*_mean_(*x*_1_, *x*_2_) model are depicted in white with the contour lines of the *f*_GI_(*x*_1_, *x*_2_) model depicted in gray: due to the white contour lines being more contracted to the origin than the gray ones, the response surface of the explicit *f*_mean_(*x*_1_, *x*_2_) model has a steeper decrease than the implicit *f*_GI_(*x*_1_, *x*_2_) model. This is also in line with the negative bias values, which are larger for the *f*_mean_(*x*_1_, *x*_2_) model in absolute terms. Thus, if the *f*_GI_(*x*_1_, *x*_2_) model spans a surface below the measured data, the *f*_mean_(*x*_1_, *x*_2_) model then definitely spans a surface below the *f*_GI_(*x*_1_, *x*_2_) model and therefore below the measured data.

**Figure 6:**
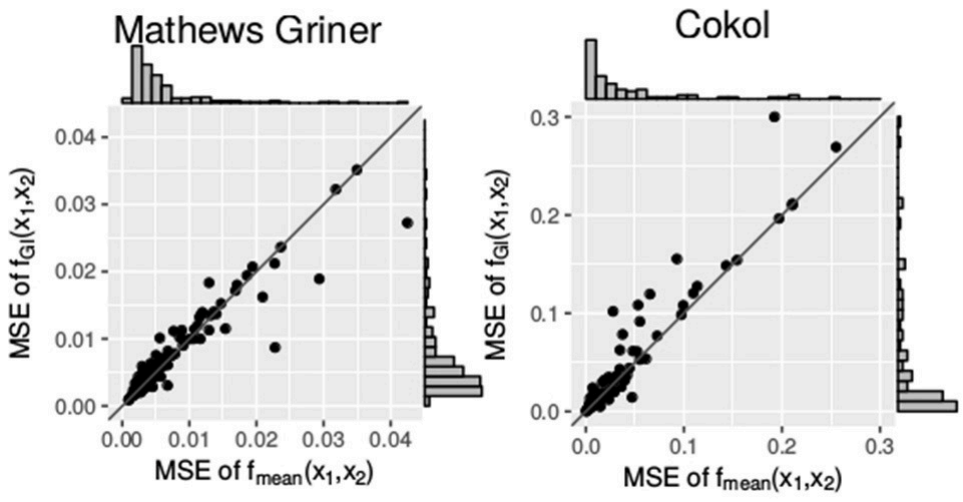
Mean squared error between the measured and the expected responses of the *f*_mean_(*x*_1_, *x*_2_) and *f*_GI_(*x*_1_, *x*_2_) model, each drawn in the according axes. To better qualify the differences in mean squared error, the diagonal is depicted. The distribution of the models' mean squared error values is given in histograms plotted on the axes.

Additionally, we compare the mean squared errors of the *f*_GI_(*x*_1_, *x*_2_) model and the *f*_mean_(*x*_1_, *x*_2_) model. We do so using the Wilcoxon signed-rank test for paired samples. For the Mathews Griner and the Cokol data, the results of the Wilcoxon signed-rank tests are significant to reject the null hypothesis of an equal mean for the alternative hypothesis that the mean squared error values of the *f*_GI_(*x*_1_, *x*_2_) model are greater. The *p*-value for the mean squared error values of both null models on the Mathews Griner data is $p_{\text{Mathews Griner}}=1.46\times 10^{-6}$ and for the Cokol data is $p_{\text{Cokol}}=7.68\times 10^{-5}$. Further, in Figure 6, the mean squared error values of both data sets are depicted in two scatter plots, the Mathews Griner data on the left and the Cokol data on the right hand side. The mean squared errors of the *f*_mean_(*x*_1_, *x*_2_) model are drawn on the *x*-axis and the mean squared errors of the *f*_GI_(*x*_1_, *x*_2_) model on the *y*-axis. The models are considered to perform equally well if their mean squared error values are equal and therefore lie on the diagonal axis. As visual aid, this diagonal is drawn in both scatter plots. Points depicted in the lower triangle of a scatter plot represent records for which the *f*_GI_(*x*_1_, *x*_2_) model results in smaller mean squared error values and points in the upper triangle represent records where the *f*_mean_(*x*_1_, *x*_2_) model performs better. There are a few outliers depicted in the lower triangle of the mean squared error values of the Mathews Griner data for records which yield a mean squared error value above 0.03 with the *f*_mean_(*x*_1_, *x*_2_) model. Investigating these records reveals a huge difference in slope parameters of the conditional responses. To give an example, the slope parameters of the record that results in a mean squared error value above 0.04 for the *f*_mean_(*x*_1_, *x*_2_) model are *s*_1_ = 1.2 and *s*_2_ = 9.6. The *f*_1→2_(*x*1, *x*2) model gives an almost ten times higher mean squared error, which is caused by the surface being strongly contracted to the origin. The majority of mean squared error values scatter in the range of [0, 0.01] and are slightly above the diagonal. In the scatter plot on the left-hand side of Figure 6, there is a clear tendency of records to scatter in the upper triangle, supporting the Wilcoxon signed-rank test result of the *f*_GI_(*x*_1_, *x*_2_) model resulting in larger mean squared error values.

### 3.3. Computational complexity

To further investigate the difference between the implicit and explicit formulation derived from the Loewe Additivity principle, we conduct a small benchmarking test. We compare the computation time of the null reference models *f*_GI_(*x*_1_, *x*_2_) (Equation 3) and *f*_mean_(*x*_1_, *x*_2_) (Equation 13). For this, we use the data set from a study conducted by Yonetani and Theorell (1964) which is believed to have no synergistic or antagonistic effect (Chou and Talalay, 1984). The data represents the inhibition of horse liver alcohol dehydrogenase by two inhibitors, ADP ribose and ADP. The data was used in an analysis of Chou and Talalay (1977). We fit the conditional parameters as described in Supplementary Material 5. For benchmarking, we use the microbenchmark package (Mersmann, 2015). It runs each calculation per default 100 times. The median time to compute the explicit formulation of Loewe Additivity is 280 times faster than the implicit one [comparing to *f*_GI_(*x*_1_, *x*_2_)]. Both, *f*_2→1_(*x*1, *x*2) and *f*_1→2_(*x*1, *x*2), have to be computed. Further results for the benchmark test on the null reference models are shown in Figure S1 in Supplementary Material 6.

## 4. Discussion

With the rise of high-throughput methods, there is a huge opportunity to investigate compound combinations for synergistic effects. Especially with a first success in a synergy study on *in vivo* mice (Grüner et al., 2016), there is an urge to develop reliable methods to screen for promising combinations. Loewe Additivity is one of the most popular principles to investigate synergistic effects in compound combination studies. With the mathematical formulation in the first part of this study we are to our knowledge the first to have developed the theoretical background and the consistency condition of Loewe Additivity. Further, this mathematical derivation led to an explicit formulation of Loewe Additivity which underlines the arbitrariness of models derived from the Loewe Additivity principle. As commented upon before (Loewe, 1928; Geary, 2012; Tallarida, 2012), we showed in two data sets that the LACC is often violated. These violations lead to differing predictions for different null reference models. This fact is generally ignored in the literature or even contradicted (Berenbaum, 1985).

Despite the common violation of the LACC, the general isobole equation is popular. Therefore, it is important to tackle the biological question of which interaction to expect in the case the LACC is violated. We introduced the explicit mean equation which is equivalent to the general isobole equation under the LACC and spans a similar surface if the LACC is violated.

Most of the synergy analyses focus on a difference in shape of isoboles at fixed effects, such as the *EC*50 effect, which, assuming a Hill curve for conditional response curves, is the parameter *e* in this research. Methods, such as the Combination Index from Berenbaum (1977) or the Median Effect Method from Chou and Talalay (1984) build upon the assumption of linear isoboles and have found wide application (Gennings et al., 2005; Sørensen et al., 2007; Cokol et al., 2011; Chevereau and Bollenbach, 2015; Chandrasekaran et al., 2016). We want to emphasize that the argument to fix the isobole to be linear by making the effect-equivalent doses $x_{1}^{\text{equiv}}$ (*x*_2_) and $x_{2}^{\text{equiv}}$ (*x*_1_) from Equations (4, 5) to be dependent on *y*, and therefore loosening the LACC in Equation (8) to a local area, namely the isobole of the effect *y*, leads to a circular type of reasoning and does not solve the ambiguity of the Loewe Additivity if the LACC does not hold.

In two non-interactive high-throughput data sets we found our new explicit mean equation null reference model to show smaller bias values than those of the general isobole equation model. This is a consequence of the more contracted surface to the origin if the LACC is violated. Further, we found the explicit mean equation to have smaller mean squared errors than the general isobole equation. These findings provide for an explicit model to replace the standard implicit model, both based on the Loewe Additivity principle. Additionally, the explicit model speeds up the computation time by a factor of roughly 250. In a large high-throughput experiment with 10,000 records this would reduce computing time from 20 h to less than 5 min. We herewith provide a first step into the direction of improving the biological and numerical issues that follow from the Loewe Additivity principle.

## Author contributions

TD conceived the research. SL performed the data analysis. All authors wrote or contributed to the writing of the manuscript.

### Conflict of interest statement

The authors declare that the research was conducted in the absence of any commercial or financial relationships that could be construed as a potential conflict of interest. The reviewer NS and handling editor declared their shared affiliation.
