# Supplementary material for: Additive Dose Response Models: Explicit Formulation and the Loewe Additivity Consistency Condition
Source: Front Pharmacol. 2018 Feb 6;9:31. doi: 10.3389/fphar.2018.00031 (PMC5808155; doi:10.3389/fphar.2018.00031)
Supplement: Supplementary file 1 [file Presentation1.pdf]

Supplementary Material: Additive Dose Response Models: Explicit Formulation and the Loewe Additivity Consistency Condition

Simone Lederer*, Tjeerd H.M. Dijkstra and Tom Heskes
*Correspondence:
Author Name: Simone Lederer
slederer@cs.ru.nl

1 LOEWE ADDITIVITY CONSISTENCY CONDITION

THEOREM 1. *If and only if a dose and its equivalent are proportional to each other (Eq. 9 and 10) the Loewe Additivity Consistency Condition in Eq. 8 holds.*

PROOF. For notational convenience we define $g \equiv f_1^{-1} \circ f_2$ thus $g^{-1} = f_2^{-1} \circ f_1$ The function g maps dose x_2 to its effect-equivalent dose x_1. The LACC can now be written as:

$$x_1 + g\left(x_2\right) = g\left(g^{-1}\left(x_1\right) + x_2\right). \tag{S1}$$

" $\Longrightarrow$ " We first provide a proof for the LACC to hold if the equivalent doses are proportional, meaning

$$x_1^{\text{equiv}}\left(x_2\right) = g\left(x_2\right) = c x_2, \tag{S2}$$

and thus

$$x_2^{\text{equiv}}\left(x_1\right) = g^{-1}\left(x_1\right) = \frac{1}{c} x_1. \tag{S3}$$

We therefore have, by rewriting Eq. S1,

$$x_1 + c x_2 \stackrel{!}{=} g\left(\frac{1}{c} x_1 + x_2\right) = c\left(\frac{1}{c} x_1 + x_2\right) = x_1 + c x_2.$$

" $\Longleftarrow$ " Proving the theorem in the other direction, we assume that the LACC holds. Starting from the statement in Eq. S1 we define the function $h\left(x_1, x_2\right)$ as the difference of these two equations:

$$h\left(x_1, x_2\right) = x_1 + g(x_2) - g(g^{-1}(x_1) + x_2) \stackrel{!}{=} 0 \qquad \forall x_1, x_2. \tag{S4}$$

As $h\left(x_1, x_2\right)$ is constant for all x_1, x_2, its first derivative has to be zero:

$$\frac{\partial h\left(x_1, x_2\right)}{\partial x_2} = g'\left(x_2\right) - g'\left(g^{-1}\left(x_1\right) + x_2\right) \stackrel{!}{=} 0. \tag{S5}$$

To further exclude that the inverse of g on x_1 is always equal to zero, we take the derivative with respect to x_1, which yields

$$\frac{\partial^2 h\left(x_1, x_2\right)}{\partial x_1 \partial x_2} = g''\left(g^{-1}\left(x_1\right) + x_2\right) \times \frac{\partial g^{-1}\left(x_1\right)}{\partial x_1} \stackrel{!}{=} 0 \qquad \forall x_1, x_2, \tag{S6}$$

from which one can deduct that $g''\left(g^{-1}\left(x_1\right) + x_2\right)$ has to be zero for all x_1, x_2. This implies that $g\left(x_2\right)$ is linear in x_2. Thus:

$$g'(x_2) = \text{constant} \quad \Rightarrow \quad g(x_2) = c_0 + cx_2$$

and since $g(0) = 0$, one obtains $c_0 = 0$. Substituting this result in Eq. 4, one gets:

$$x_1^{\text{equiv}}\left(x_2\right) = g(x_2) = cx_2, \tag{S7}$$

which shows equivalent doses to be proportional.

2 GENERAL ISOBOLE EQUATION UNDER LACC

COROLLARY 1. *If the Loewe Additivity Consistency Condition in Eq. 8 holds,* (1) $f_{GI}\left(x_1, x_2\right) = f_{2\to 1}\left(x_1, x_2\right) = f_{1\to 2}\left(x_1, x_2\right)$ *and* (2) *the isoboles are parallel.*

PROOF. (1) Assume a response level denoted by y^*, then the doses of compounds 1 and 2 that cause this effect by themselves are given by $f_1^{-1}(y^*)$ and $f_2^{-1}(y^*)$ being related by concentration scaling, i.e. $f_1^{-1}\left(y^*\right) = cf_2^{-1}\left(y^*\right)$ (Eq. 9 and 10). The isobole for effect y^* is given by

$$\begin{aligned} y^* &= f_1(x_1 + cx_2) \\ \Rightarrow \quad f_1^{-1}(y^*) &= x_1 + \frac{f_1^{-1}(y^*)}{f_2^{-1}(y^*)} x_2, \end{aligned}$$

which leads to the linear isobole equation depicted in Eq. 3, assuming f is a continuous and monotonic response:

$$\frac{x_1}{f_1^{-1}(y^*)} + \frac{x_2}{f_2^{-1}(y^*)} = 1. \tag{S8}$$

(2) For a given y^*, this linear isobole equation gives the contour lines of the response surface. Since the two concentrations x_1^* and x_2^* are related by linear scaling ($x_1^* = x_1^{\text{equiv}}\left(x_2\right) = cx_2^*$, see Eq. 9), the isoboles for different effect levels y^* are parallel. This becomes clear by replacing x_2^* with its equivalent cx_1^* in the linear isobole equation and solving then for x_2:

$$\begin{aligned} \frac{x_1}{x_1^*} + \frac{x_2}{x_2^*} &= \frac{x_1}{x_1^*} + \frac{x_2}{cx_1^*} = 1 \\ \Leftrightarrow \qquad x_2 &= cx_1^* - cx_1 = c\left(x_1^* - x_1\right), \end{aligned}$$

where $x_1^* = f_1^{-1}\left(y^*\right)$. Therefore, x_2 is linearly dependent with a fixed slope parameter $c > 0$. This results in an isobole with slope $-c$ for any effect y^* that is reached by x_1^*, and therefore the isoboles are parallel.

3 CONSISTENCY CONDITION FOR HILL CURVE

COROLLARY 2. *If the* Loewe Additivity Consistency Condition *in Eq. 8 holds with f_1 and f_2 taking the form of two Hill curves, then the slopes and effect ranges y_0 and y_∞ of the Hill curves must be the same. Further, the proportionality factor c takes the form of a fraction of the EC$_{50}$ value of the drug to be expressed in terms of the other divided by the EC$_{50}$ value of this other drug, resulting in $x_1^{equiv}(x_2) = \frac{e_1}{e_2}x_2$.*

For the Loewe Additivity Consistency Condition,

$$x_1^{\text{equiv}}(x_2) = f_1^{-1}(f_2(x_2)) = cx_2, \tag{S9}$$

to be fulfilled when using the Hill curve for $f_j, j \in \{1,2\}$, which is of the form

$$f_j(x_j) = y_{\infty,j} + \frac{y_{0,j} - y_{\infty,j}}{1 + \left(\frac{x_j}{e_j}\right)^{s_j}}, \tag{S10}$$

with its inverse being of the form

$$f^{-1}(y_j) = \left[\frac{y_{0,j} - y_j}{y_j - y_{\infty,j}}\right]^{1/s_j} e_j \tag{S11}$$

one gets:

$$\begin{aligned}
x_1^{\text{equiv}}(x_2) =& f_1^{-1}(f_2(x_2)) \\
=& \left[\frac{y_{0,1} - y_{\infty,1}}{f_2(x_2) - y_{\infty,1}} - 1\right]^{1/s_1} e_1 \\
=& \left[\frac{y_{0,1} - y_{\infty,1}}{y_{\infty,2} + \frac{y_{0,2} - y_{\infty,2}}{1 + \left(\frac{x_2}{e_2}\right)^{s_2}} - y_{\infty,1}} - 1\right]^{1/s_1} e_1 \\
=& \left[\frac{y_{0,1} - y_{\infty,1} + (y_{0,1} - y_{\infty,1})\left(\frac{x_2}{e_2}\right)^{s_2}}{y_{0,2} - y_{\infty,1} + (y_{\infty,2} - y_{\infty,1})\left(\frac{x_2}{e_2}\right)^{s_2}} - 1\right]^{1/s_1} e_1 \\
=& \left[\frac{y_{0,1} - y_{0,2} + (y_{0,1} - y_{\infty,2})\left(\frac{x_2}{e_2}\right)^{s_2}}{y_{\infty,1} - y_{0,2} + (y_{\infty,1} - y_{\infty,2})\left(\frac{x_2}{e_2}\right)^{s_2}}\right]^{1/s_1} e_1
\end{aligned}$$

Hence, in order for $x_1^{\text{equiv}}(x_2)$ to be constant $y_{\infty,1} = y_{\infty,2}$, which gives:

$$x_1^{\text{equiv}}(x_2) = \left[\frac{y_{0,1} - y_{0,2} + (y_{0,1} - y_\infty)\left(\frac{x_2}{e_2}\right)^{s_2}}{y_\infty - y_{0,2}}\right]^{1/s_1} e_1$$

and $y_{0,1} = y_{0,2}$, simplifying to:

$$\begin{aligned} x_1^{\text{equiv}}(x_2) &= \left[\frac{(y_0 - y_\infty)\left(\frac{x_2}{e_2}\right)^{s_2}}{y_\infty - y_0}\right]^{1/s_1} e_1 \\ &= -\left(\frac{x_2}{e_2}\right)^{s_2/s_1} e_1 \\ &= x_2^{s_2/s_1}\frac{e_1}{e_2^{s_2/s_1}} \end{aligned}$$

which is constant if $s_2/s_1 = 1 \Leftrightarrow s_1 = s_2$.

The Loewe Additivity Consistency Condition is therefore fulfilled for the Hill curves in the same ranges, $y_{\infty,1} = y_{\infty,2}$ and $y_{0,1} = y_{0,2}$ and with the same slope $s_1 = s_2$.

4 VIOLATION OF THE LOEWE ADDITIVITY CONSISTENCY CONDITION

When the LACC applies, the General Isobole Equation, $f_{\text{GI}}(x_1, x_2)$, and both explicit solutions, $f_{2\to1}(x_1, x_2)$ and $f_{1\to2}(x_1, x_2)$, are equivalent. When the LACC fails, one may wonder how to combine the two explicit solutions such that they are still close to the solution of the General Isobole Equation Eq. 3. Since the solution of the General Isobole Equation Eq. 3 is symmetric, it makes sense to take the (weighted) mean of the two solutions $f_{2\to1}(x_1, x_2)$ and $f_{1\to2}(x_1, x_2)$. Therefore, we define

$$f_{\text{mean}}(x_1, x_2) = \beta(x_1, x_2) f_{2\to1}(x_1, x_2) + [1 - \beta(x_1, x_2)] f_{1\to2}(x_1, x_2)\ , \qquad \text{(S12)}$$

with $\beta(x_1, x_2)$ a weighting function. We wonder how to choose $\beta(x_1, x_2)$ such that $f_{\text{mean}}(x_1, x_2) \approx f_{\text{GI}}(x_1, x_2)$ under mild violations of the LACC.

In case the LACC holds, we have

$$x_1^{\text{equiv}}(x_2) = c x_2 \ \text{ and } \ x_2^{\text{equiv}}(x_1) = \frac{x_1}{c}\ ,$$

i.e. the equivalent doses are proportional to the original doses. To study mild violations of LACC, which we will refer to as LACC-ϵ, we add a small quadratic term, i.e., consider

$$x_1^{\text{equiv}}(x_2) = c x_2 + \epsilon c x_2^2 \ \text{ and } \ x_2^{\text{equiv}}(x_1) = \frac{x_1}{c} - \epsilon \frac{x_1^2}{c^2}\ ,$$

such that we have the consistency equations:

$$x_1^{\text{equiv}}(x_2^{\text{equiv}}(x_1)) = x_1 + \mathcal{O}(\epsilon^2)$$

and

$$x_2^{\text{equiv}}(x_1^{\text{equiv}}(x_2)) = x_2 + \mathcal{O}(\epsilon^2)$$

where we take ϵ to be small so that we can ignore all second and higher order terms in ϵ. Note the different dependence on c in the two quadratic terms. These are necessary for the consistency condition to hold. Our goal is now to find $\beta(x_1, x_2)$ such that under LACC-ϵ we still have $f_{\text{mean}}(x_1, x_2) = f_{\text{GI}}(x_1, x_2)$, or, to put it differently, such that $f_{\text{mean}}(x_1, x_2)$ still satisfies the General Isobole Equation.

THEOREM 2. *Under LACC-ϵ, the simple arithmetic mean $f_{mean}(x_1, x_2)$ with $\beta(x_1, x_2) = 1/2$ satisfies the General Isobole Equation.*

PROOF. As an intermediate step, we note that we can rewrite f_2 into f_1 and vice versa through

$$f_2(x_2 + x_2^{\text{equiv}}(x_1)) = f_1(x_1^{\text{equiv}}(x_2 + x_2^{\text{equiv}}(x_1)) \text{ and}$$
$$f_1(x_1 + x_1^{\text{equiv}}(x_2)) = f_2(x_2^{\text{equiv}}(x_1 + x_1^{\text{equiv}}(x_2)) \, ,$$

with

$$\begin{aligned} x_1^{\text{equiv}}&(x_2 + x_2^{\text{equiv}}(x_1)) = \\ &= cx_2 + cx_2^{\text{equiv}}(x_1) + \epsilon c(x_2 + x_2^{\text{equiv}}(x_1))^2 \\ &= cx_2 + x_1 - \epsilon\frac{x_1^2}{c} + \epsilon c x_2^2 + 2\epsilon x_1 x_2 + \epsilon\frac{x_1^2}{c} \\ &= x_1 + x_1^{\text{equiv}}(x_2) + 2\epsilon x_1 x_2 \, . \end{aligned}$$

and

$$\begin{aligned} x_2^{\text{equiv}}&(x_1 + x_1^{\text{equiv}}(x_2)) = \\ &= \frac{x_1}{c} + \frac{x_1^{\text{equiv}}(x_1)}{c} - \epsilon\frac{(x_1 + x_1^{\text{equiv}}(x_2))^2}{c^2} \\ &= \frac{x_1}{c} + x_2 + \epsilon x_2^2 - \epsilon\frac{x_1^2}{c^2} - 2\epsilon\frac{x_1 x_2}{c} - \epsilon x_2^2 \\ &= x_2 + x_2^{\text{equiv}}(x_1) - 2\epsilon\frac{x_1 x_2}{c} \, , \end{aligned}$$

where, here and in the following, we ignore second and higher order terms in ϵ.

For $f_{\text{mean}}(x_1, x_2)$ to satisfy the General Isobole Equation, we need

$$\frac{x_1}{f_1^{-1}(f_{\text{mean}}(x_1, x_2))} + \frac{x_2}{f_2^{-1}(f_{\text{mean}}(x_1, x_2))} = 1 \, .$$

Luckily, using the above expressions, we have two ways to rewrite $f_{\text{mean}}(x_1, x_2)$: in terms of f_1,

$$\begin{aligned} & f_{\text{mean}}(x_1, x_2) \\ &= \beta(x_1, x_2) f_1(x_1 + x_1^{\text{equiv}}(x_2)) \\ &\quad + [1 - \beta(x_1, x_2)] f_1(x_1 + x_1^{\text{equiv}}(x_2) + 2\epsilon x_1 x_2) \\ &= f_1\left(x_1 + x_1^{\text{equiv}}(x_2) + 2\epsilon[1 - \beta(x_1, x_2)] x_1 x_2\right) , \end{aligned}$$

and in terms of f_2,

$$\begin{aligned} & f_{\text{mean}}(x_1, x_2) \\ &= \beta(x_1, x_2) f_2(x_2 + x_2^{\text{equiv}}(x_1) - 2\epsilon x_1 x_2 / c) \\ &\quad + [1 - \beta(x_1, x_2)] f_2(x_2 + x_2^{\text{equiv}}(x_1)) \\ &= f_2(x_2 + x_2^{\text{equiv}}(x_1) - 2\epsilon\beta(x_1, x_2) x_1 x_2 / c) , \end{aligned}$$

where the last step follows from the observation that, again keeping track of just the first order terms in ϵ,

$$\begin{aligned} & \beta f(x + \epsilon) + (1 - \beta) f(x) \\ &= \beta f(x) + \beta\epsilon f'(x) + (1 - \beta) f(x) \\ &= f(x) + \beta\epsilon f'(x) = f(x + \beta\epsilon) . \end{aligned} \tag{S13}$$

Plugging these two formulations at the obvious places in the General Isobole Equation, we obtain

$$\begin{aligned} 1 &= \frac{x_1}{f_1^{-1}(f_{\text{mean}}(x_1, x_2))} + \frac{x_2}{f_2^{-1}(f_{\text{mean}}(x_1, x_2))} \\ &= \frac{x_1}{x_1 + x_1^{\text{equiv}}(x_2) + 2\epsilon[1 - \beta(x_1, x_2)] x_1 x_2} \\ &\quad + \frac{x_2}{x_2 + x_1^{\text{equiv}}(x_2) - 2\epsilon\beta(x_1, x_2) x_1 x_2 / c} \\ &= \frac{x_1}{x_1 + c x_2 + \epsilon c x_2^2 + 2\epsilon[1 - \beta(x_1, x_2)] x_1 x_2} \\ &\quad + \frac{c x_2}{c x_2 + x_1 - \epsilon x_1^2 / c - 2\epsilon\beta(x_1, x_2) x_1 x_2} . \end{aligned}$$

Further expansion in ϵ yields

$$\begin{aligned} 0 &= -\frac{x_1^2}{(x_1 + c x_2)^2} \left\{ c x_2^2 + 2[1 - \beta(x_1, x_2)] x_1 x_2 \right\} \epsilon \\ &\quad + \frac{c^2 x_2^2}{(x_1 + c x_2)^2} \left\{ x_1^2 / c + 2\beta(x_1, x_2) x_1 x_2 \right\} \epsilon . \end{aligned}$$

So, for the first order term in ϵ to cancel, we must have

$$\begin{aligned} 0 &= -cx_1x_2 - 2[1-\beta(x_1,x_2)] + cx_1x_2 + 2\beta(x_1,x_2) \\ &= -2 + 4\beta(x_1,x_2) \,, \end{aligned}$$

with solution $\beta(x_1,x_2) = 1/2$: simple equal weighting.

5 DATA CLEANING, FITTING OF HILL CURVE AND PARAMETER ESTIMATION FOR IMPLICIT MODELS

A first step of processing the data includes an outlier analysis of the raw reads by fitting a spline surface and deleting outliers. Further, we explain how to calculate the implicit response values of the $f_{\mathrm{GI}}\left(x_1,x_2\right)$ model.

To detect outliers, we fit the data to a general additive model (GAM) using thin plate splines (Wood, 2017). We use the method gam() of the mgcv-package (Wood, 2016). Every data point is rejected for which its absolute residual value is larger than three times the inter-quantile range of all residuals of a given record. For the Mathews Griner data, this leads to 125 records out of the 466 where a mean of 1.59 outliers were excluded per record. A maximum of 6 outliers was detected once. Similarly, we excluded 4.21 data points for the Cokol data on 150 of the total 200 records with a maximum of 13 data points.

The two conditional responses of a record are fitted in parallel to two Hill functions in the form of Eq. 1 with the drc package (Ritz and Strebig, 2016). Unlike other synergy analyses such as (Yadav et al., 2015), the response at zero concentration y_0 is not fixed but only constrained to be the same for both response curves. The other Hill parameters, y_∞, s and e are fitted for both compounds individually.

The $f_{\mathrm{GI}}\left(x_1,x_2\right)$ model is an implicit model for the response y. Therefore, a root finder is used to find a response $\hat{y}_i$ for a given parameter set $\Theta = \{y_0, y_{\infty,j}, e_j, s_j\}$, and concentrations $x_{i,1}$ $x_{i,2}$. For finding such a root the standard implementation of a root finder in the R stats package, uniroot() (R Core Team, 2016), is used which uses the Brent-Dekker-van Wijngaarden algorithm (Press et al., 2007, Chapter 9). As convergence criterion we used 1.22×10^{-4}. To ensure the existence of the inverse values of the Hill curve at a given response y, $f^{-1}\left(y\right)$, the responses are limited to the range for which the Hill curve is defined, $[y_0, y_\infty]$. Responses outside this range are set to the closest range limits, i.e. if $y > y_0 \Rightarrow y = y_0$ and if $y < y_\infty \Rightarrow y = y_\infty$.

6 BENCHMARK TEST ON GENERATED DATA FROM YONETANI

To show the speed advantage of the explicit formulation derived from the Loewe Additivity principle, we conducted a benchmarking test. We compare the computation time of the null reference models GI (Eq. 3), $f_{2\to1}\left(x_1,x_2\right)$ (Eq. 6), $f_{1\to2}\left(x_1,x_2\right)$ (Eq. 7), and Explicit Mean Equation (Eq. 13). For this, we use the data set from Yonetani and Theorell (1964) which is known to be non-interactive (Chou and Talalay, 1984). We fit the conditional parameters as described in Supplementary Material 5. For the benchmarking test, we made use of the microbenchmark package (Mersmann, 2015). It runs each calculation per default 100 times.

A visualization of the runtime is depicted in Fig. S1 The explicit formulations are clearly faster in computing time than the implicit ones.

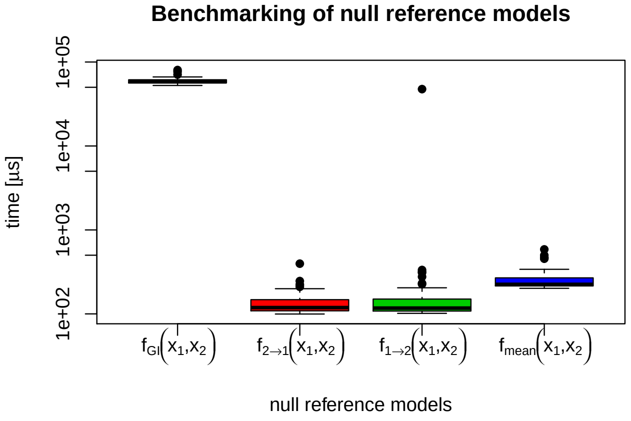

Figure S1. Benchmarking times on a log-scale for the four models $f_{\text{GI}}(x_1, x_2)$ (Eq. 3), the explicit formulation (Eq. 6 and Eq. 7), and its mean formulation $f_{\text{mean}}(x_1, x_2)$ (Eq. 13).

7 GEOMETRIC MEAN MODEL

Analogously to the weighted mean, we take the geometric mean of the two explicit models $f_{2\to 1}(x_1, x_2)$ and $f_{1\to 2}(x_1, x_2)$ depicted in Eq. 6 and 7

$$f_{\text{geometric}}(x_1, x_2) = \sqrt{f_{2\to 1} f_{1\to 2}}. \tag{S14}$$

In parallel to the Explicit Mean Equation, the geometric mean is the least sensitive to a violation of the LACC with an equal weighting of both formulations $f_{2\to 1}$ and $f_{1\to 2}$: Assume the Explicit Geometric Mean Equation model with weights $\beta(x_1, x_2)$:

$$f_{\text{geometric}}(x_1, x_2) = f_{2\to 1}(x_1, x_2)^{\beta(x_1,x_2)} f_{1\to 2}(x_1, x_2)^{1-\beta(x_1,x_2)}\ . \tag{S15}$$

Obviously, under LACC we have $f_{\text{geometric}}(x_1, x_2) = f_{\text{mean}}(x_1, x_2) = f_{\text{GI}}(x_1, x_2)$ for any choice of $\beta(x_1, x_2)$.

THEOREM 1. *Under LACC-ϵ, the (weighted) geometric mean $f_{geometric}(x_1, x_2)$ equals the arithmetic mean $f_{mean}(x_1, x_2)$ with the same $\beta(x_1, x_2)$.*

PROOF. Following the exact same line of reasoning as in the proof of Theorem 2 up to Eq. S13 in Supplementary Material 4, again ignoring second and higher order terms in ϵ, we get

$$\begin{aligned}
& f_{\text{geometric}}\left(x_1, x_2\right) \\
= \quad & f_1\left(x_1 + x_1^{\text{equiv}}(x_2)\right)^{\beta(x_1,x_2)} \\
& \times f_1\left(x_1 + x_1^{\text{equiv}}(x_2) - 2\epsilon x_1 x_2\right)^{1-\beta(x_1,x_2)} \\
= \quad & \exp\left\{\beta\left(x_1, x_2\right) \log f_1\left(x_1 + x_1^{\text{equiv}}(x_2)\right)\right. \\
& + [1 - \beta(x_1, x_2)] \\
& \left. \times \log f_1\left(x_1 + x_1^{\text{equiv}}(x_2) - 2\epsilon x_1 x_2\right)\right\} \\
= \quad & f_1(x_1 + x_1^{\text{equiv}}(x_2) + 2\epsilon[1 - \beta(x_1, x_2)]x_1 x_2) \\
= \quad & f_{\text{mean}}\left(x_1, x_2\right) \ ,
\end{aligned}$$

where we applied the reasoning of (S13) to the term in the exponent.

This then gives the following corollary:

COROLLARY 3. *Under LACC-ϵ, the simple geometric mean $f_{geometric}\left(x_1, x_2\right)$ with $\beta(x_1, x_2) = 1/2$ satisfies the General Isobole Equation.*

Analogously to Fig. 4D, the Explicit Geometric Mean Equation model takes different shapes for the different violations of LACC, as depicted in Fig. 3. They are depicted in Fig. S2

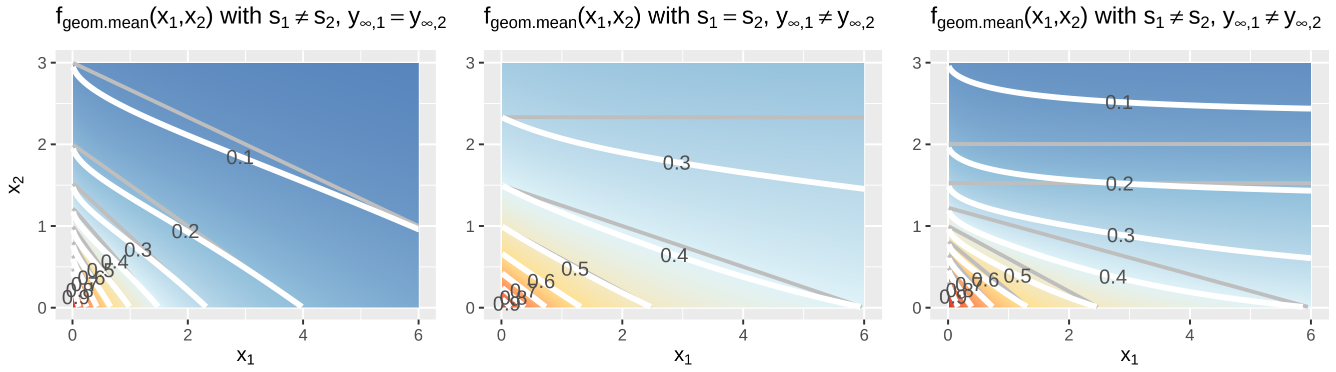

Figure S2. Contour lines of the $f_{\text{mean}}\left(x_1, x_2\right)$ model for three different scenarios, where the LACC is violated: from left to right: the slopes are different, $s_1 \neq s_2$, here depicted with $s_1 = 1, s_2 = 2, y_\infty = 0$, or the maximal effect values differ, $y_{\infty,1} = y_{\infty,2}$, here shown with $s = 1, y_{\infty,1} = 0.3, y_{\infty,2} = 0$ or both are different, here shown with $s_1 = 1, s_2 = 2, y_{\infty,1} = 0.3, y_{\infty,2} = 0$. The remaining two parameters of the Hill curve are set equally for all figures to $y_0 = 1$ and $e = 1$ are equal.

We compute the mean squared error values of each non-interactive record and compare them with the mean squared errors of the $f_{\text{GI}}\left(x_1, x_2\right)$ model. The scatter plots for the two data sets are depicted in Fig. S3. A Wilcoxon signed-rank on both error value sets for the $f_{\text{GI}}\left(x_1, x_2\right)$ and the $f_{\text{geometric}}\left(x_1, x_2\right)$ model, with

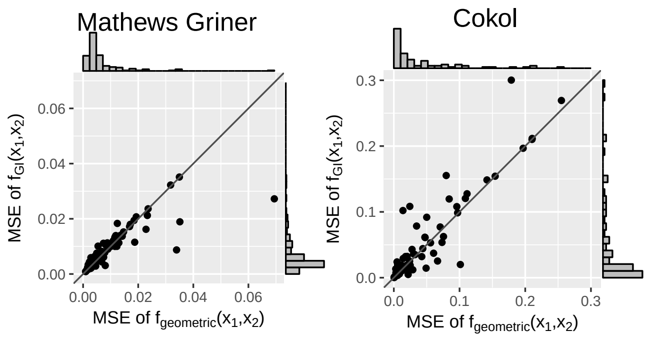

Figure S3. Mean squared error between the measured and the expected responses of the $f_{\mathrm{geometric}}\,(x_1, x_2)$ and $f_{\mathrm{GI}}\,(x_1, x_2)$ model. To better qualify the differences in mean squared error, the diagonal is depicted. The distribution of the models' mean squared error is given in histograms plotted on the axes.

the alternative hypothesis being that the errors of $f_{\mathrm{GI}}\,(x_1, x_2)$ are larger, give the following p-values: for the Mathews Griner data set: 6.38×10^{-6} and for Cokol 7.26×10^{-4}.

REFERENCES

Chou, T.-C. and Talalay, P. (1984). Quantitative analysis of dose-effect relationships: the combined effects of multiple drugs or enzyme inhibitors. *Adv. Enzyme Regul.* 22, 27–55. doi:10.1016/0065-2571(84)90007-4

Mersmann, O. (2015). *microbenchmark: Accurate Timing Functions*

Press, W. H., Teukolsky, S. a., Vetterling, W. T., and Flannery, B. P. (2007). *Numerical Recipes: The Art of Scientific Computing*, vol. 1 (Cambridge: Cambridge University Press), 3 edn. doi:10.1137/1031025

R Core Team (2016). *R: A Language and Environment for Statistical Computing*. R Foundation for Statistical Computing, Vienna, Austria

Ritz, C. and Strebig, J. C. (2016). *drc: Analysis of Dose-Response Curves*

Wood, S. (2016). *mgcv: Mixed GAM Computation Vehicle with GCV/AIC/REML Smoothness Estimation*

Wood, S. (2017). *Generalized Additive Models: an introduction with R* (Chapman and Hall/CRC), 2 edn. doi:10.1111/j.1541-0420.2006.00574.x

Yadav, B., Wennerberg, K., Aittokallio, T., and Tang, J. (2015). Searching for Drug Synergy in Complex Dose-Response Landscapes Using an Interaction Potency Model. *Comput. Struct. Biotechnol. J.* 13, 504–513. doi:10.1016/j.csbj.2015.09.001

Yonetani, T. and Theorell, H. (1964). Studies on liver alcohol dehydrogenase complexes: III. Multiple inhibition kinetics in the presence of two competitive inhibitors. *Arch. Biochem. Biophys.* 106, 243–251. doi:10.1016/0003-9861(64)90184-5
